# Accuracy and Usability of a New Rapid Diagnostic Test for the Diagnosis of Dengue in Vientiane, Laos

**DOI:** 10.1093/ofid/ofag422

**Published:** 2026-07-14

**Authors:** Vilayouth Phimolsannousith, Bountoy Sibounheuang, Anisone Chanthongthip, Manivanh Vongsouvath, Mayfong Mayxay, Mavuto Mukaka, Manophab Luangraj, Paul N Newton, Elizabeth A Ashley, Heidi Hopkins, Audrey Dubot-Pérès

**Affiliations:** Lao–Oxford–Mahosot Hospital–Wellcome Trust Research Unit, Microbiology Laboratory, Mahosot Hospital, Vientiane, Lao People’s Democratic Republic; Lao–Oxford–Mahosot Hospital–Wellcome Trust Research Unit, Microbiology Laboratory, Mahosot Hospital, Vientiane, Lao People’s Democratic Republic; Lao–Oxford–Mahosot Hospital–Wellcome Trust Research Unit, Microbiology Laboratory, Mahosot Hospital, Vientiane, Lao People’s Democratic Republic; Lao–Oxford–Mahosot Hospital–Wellcome Trust Research Unit, Microbiology Laboratory, Mahosot Hospital, Vientiane, Lao People’s Democratic Republic; Lao–Oxford–Mahosot Hospital–Wellcome Trust Research Unit, Microbiology Laboratory, Mahosot Hospital, Vientiane, Lao People’s Democratic Republic; Centre for Tropical Medicine and Global Health, Nuffield Department of Clinical Medicine, University of Oxford, Oxford, United Kingdom; Institute of Research and Education Development, University of Health Sciences, Vientiane, Lao People’s Democratic Republic; Saw Swee Hock School of Public Health, National University of Singapore, Singapore, Singapore; Centre for Tropical Medicine and Global Health, Nuffield Department of Clinical Medicine, University of Oxford, Oxford, United Kingdom; Mahidol Oxford Tropical Medicine Research Unit, Faculty of Tropical Medicine, Mahidol University, Bangkok, Thailand; Lao–Oxford–Mahosot Hospital–Wellcome Trust Research Unit, Microbiology Laboratory, Mahosot Hospital, Vientiane, Lao People’s Democratic Republic; Lao–Oxford–Mahosot Hospital–Wellcome Trust Research Unit, Microbiology Laboratory, Mahosot Hospital, Vientiane, Lao People’s Democratic Republic; Centre for Tropical Medicine and Global Health, Nuffield Department of Clinical Medicine, University of Oxford, Oxford, United Kingdom; Mahidol Oxford Tropical Medicine Research Unit, Faculty of Tropical Medicine, Mahidol University, Bangkok, Thailand; Faculty of Infectious and Tropical Diseases, London School of Hygiene and Tropical Medicine, London, United Kingdom; Lao–Oxford–Mahosot Hospital–Wellcome Trust Research Unit, Microbiology Laboratory, Mahosot Hospital, Vientiane, Lao People’s Democratic Republic; Centre for Tropical Medicine and Global Health, Nuffield Department of Clinical Medicine, University of Oxford, Oxford, United Kingdom; Faculty of Infectious and Tropical Diseases, London School of Hygiene and Tropical Medicine, London, United Kingdom; Lao–Oxford–Mahosot Hospital–Wellcome Trust Research Unit, Microbiology Laboratory, Mahosot Hospital, Vientiane, Lao People’s Democratic Republic; Centre for Tropical Medicine and Global Health, Nuffield Department of Clinical Medicine, University of Oxford, Oxford, United Kingdom; Unité des Virus Émergents, Aix-Marseille Université, Università di Corsica, Institute of Research for Development 190, Inserm 1207, IRBA (Institut de Recherche Biomédicale des Armées), Marseille, France

**Keywords:** dengue, diagnostic accuracy, immunochromatographic rapid diagnostic tests, Lao PDR

## Abstract

**Background:**

An estimated 390 million dengue virus (DENV) infections occur per year, 70% in the Western Pacific and Southeast Asia. A prototype rapid diagnostic test (RDT) (NS1, immunoglobulin M [IgM]/immunoglobulin G [IgG]; DengueDx, Mologic) was evaluated for accuracy and ease of use for dengue diagnosis at Mahosot Hospital, Vientiane, Lao People’s Democratic Republic.

**Methods:**

Five hundred twenty-six sera collected in 2017 and 2018 from patients hospitalized with suspected dengue were included. Prototype RDT results were compared to NS1 and IgM enzyme-linked immunosorbent assays and/or real-time reverse-transcription polymerase chain reaction as reference assays and to Bioline dengue RDT (NS1, IgM/IgG) as comparator. The accuracy of the image-based result interpretation (RDT scan or photograph) at day 0 and after a delay of 7 days was evaluated. Five technicians were observed when performing RDTs and interviewed on their perception of the ease of use.

**Results:**

The overall sensitivity and the sensitivity for NS1 detection alone were significantly lower for the prototype RDT (65.2% [95% confidence interval {CI}, 59.3%–70.8%] and 44.6% [95% CI, 38.0%–51.4%], respectively) than for the Bioline RDT (76.7% [95% CI, 69.1%–83.2%], z-test *P* = .014; and 66.7% [95% CI, 57.5%–75.0%], z-test *P* < .001, respectively). The positive predictive value of the prototype RDT for detection of NS1 was >90%. Only half of the positive results were found positive when reading results from images. Correlation of >98% for positive results was obtained between reading at day 0 and reading at day 7. During ease of use assessment, when performing the Bioline RDTs the participants made fewer errors and were more satisfied than when performing the prototype RDTs.

**Conclusions:**

Improvement in test sensitivity and in device design would adapt the prototype RDT for Laos and similar contexts. Optimization of the prototype RDT is warranted and further evaluation in different healthcare settings would provide a more accurate estimation of the value of this RDT for dengue surveillance in remote areas.

Dengue virus (DENV), an enveloped single-stranded RNA virus, is transmitted by *Aedes* mosquitoes. Infection by any of the 4 dengue serotypes (DENV-1 to -4) can cause disease from mild dengue fever to severe dengue hemorrhagic fever and dengue shock syndrome [1]. DENV transmission is hyperendemic in tropical and subtropical areas, with an estimated 390 million infections per year [2]. Nearly 4 billion people are considered at risk of contracting dengue, of whom 70% live in the Western Pacific or Southeast Asia [3]. In Lao People's Democratic Republic (hereafter “Laos”), dengue infection is a major cause of morbidity with increasing disease burden [4]. Approximately 3.9 million residents are presently at risk of dengue infection. It is usually regarded as an urban disease, but recent studies in Laos suggest that it is also an important rural disease [5–8]. However, only limited robust data on dengue epidemiology in Laos are available. Only a few institutions, located in the capital city (Vientiane), have access to laboratory facilities required to perform enzyme-linked immunosorbent assay (ELISA) and reverse-transcription polymerase chain reaction (RT-PCR), and transportation of frozen specimens is difficult. An alternative diagnostic tool is immunochromatographic rapid diagnostic tests (RDTs). Although RDTs are rolled out widely in the country, the accuracy of the RDTs used is not always well evaluated.

RDTs are commonly used at the point of care to diagnose primary and post-primary dengue infection. By their format, RDTs satisfy many of the World Health Organization ASSURED criteria (Affordable, Sensitive, Specific, User-friendly, Rapid, Equipment-free, and Delivered). They are rapid, easy to use, do not require specific technical knowledge or equipment, and have shown moderately good accuracy in endemic areas, especially for nonstructural protein 1 (NS1) detection during the acute phase of infection, with reported pooled sensitivity of 70% and pooled specificity of 95.4% [9]. The Standard Diagnostics (SD) Bioline Dengue Duo RDT (Bioline RDT; Standard Diagnostic, Kyoggi-do, Korea) for the detection of both dengue virus NS1 antigen and anti-DENV immunoglobulin M (IgM)/immunoglobulin G (IgG) antibodies has been used in multiple studies showing overall satisfactory accuracy (sensitivity from 80.7% to 97.2% and specificity from 72.7% to 100%) for dengue diagnosis [9–15]. However, it costs around 3 US dollars (USD) per test, which makes it unaffordable for most hospitals in Laos, especially in rural areas, thereby failing to meet the first ASSURED criterion.

Mologic, Ltd [16] developed a prototype dengue RDT called DengueDx (Global Access Dx [formerly Mologic], Bedford, UK) for combined detection of NS1 and IgM/IgG, which integrates virus-like particle technology [17]; requires low sample volumes of blood, plasma, or serum (10 μL for IgM/IgG testing and 40 μL for NS1 testing); and is manufactured at cost <1 USD per test with plans to supply to low-income settings at cost of goods, thereby fulfilling the first ASSURED criterion.

Preliminary accuracy data provided by the manufacturer suggested good sensitivity and specificity compared to various reference standards (unpublished data, personal communication, Mologic February 2020). Before implementation in clinical practice or surveillance, RDT performance must be evaluated independently in the epidemiological and clinical contexts where it is intended for use in diagnosis.

To evaluate whether the prototype RDT meets all ASSURED criteria, we assessed its diagnostic accuracy and ease of use for dengue diagnosis in Laos, using a panel of sera from inpatients at Mahosot Hospital suspected to have dengue fever. The results obtained from the prototype RDT were compared with the results obtained for the same panel of sera tested by probe-based real-time RT-PCR (qRT-PCR), NS1 ELISA, and/or IgM ELISA, as reference standards and with the Bioline dengue RDT.

## MATERIALS AND METHODS

### Study Design

This was a retrospective observational case series study, reported here following the Standards for the Reporting of Diagnostic Accuracy Studies (STARD) guidelines (Checklist S1) [18].

### Samples

In 2017 and 2018, blood samples from patients admitted to Mahosot Hospital, Vientiane Capital, Laos, and whom the ward physician suspected of having dengue infection based on clinical signs and symptoms, were sent for routine dengue diagnosis at the virology laboratory in Mahosot Hospital. For each patient, admission serum was tested by pan-DENV probe-based qRT-PCR, as previously described [19], and SD Bioline Dengue Duo (dengue NS1 antigen and IgG/IgM) (Bioline RDT; Abbott, IL, USA, catalog number 11FK46). Bioline RDTs were performed at the time of sample collection and read by a laboratory technician experienced in performing and reading RDTs. Leftover serum was immediately stored at −80°C; all consecutive samples for which enough volume was available for the prototype DengueDx RDT and NS1 and IgM ELISAs were included in the current study. Those assays were performed in 2021.

### Prototype RDT

The sera were thawed and immediately tested using the prototype RDTs from a single batch (DNLFD-006-1 for NS1 and DILFD-005-2 for IgM/IgG), following the manufacturers’ instructions, which are briefly described below, with the exception that the samples were loaded onto RDTs using a micropipette rather than the blood transfer device provided in test kits (to ensure reproducibility of sample volume loaded). The tests were prepared and read by laboratory technicians experienced in performing and reading RDTs.

The prototype RDT consists of 2 cassettes: 1 single-test-strip cassette detecting NS1 antigen, and 1 dual-test-strip cassette detecting IgM and IgG anti-DENV antibodies. For the NS1 cassette, 40 μL of serum was loaded on the sample well. After 30 seconds, 1 drop of buffer was added to the sample well by squeezing the provided bottle of buffer. The result was read after 10 minutes of incubated at ambient temperature. A red line appears at the NS1 test line level in case of detection of DENV NS1. The result (negative or positive) is valid only if a red line appears at the control line level.

For the antibody cassette, 10 μL of serum was loaded on the sample well. After 30 seconds, 2 drops of buffer were added to the buffer well by squeezing the provided bottle of buffer. The result was read after 10 minutes of incubation at ambient temperature. A red line appears at the IgM test line level in case of detection of anti-DENV IgM, and a red line at the IgG test line in case of detection of anti-DENV IgG. The result (negative or positive) is valid only if a red line appears at the control line level.

Each RDT result was read by 2 independent readers. A third independent reader read the RDT at the same time as the 2 other readers, but those results were used only to resolve cases of discrepancy between the 2 other readers. All 3 readers read the RDTs immediately after completion of the 10-minute test development time, blinded to each other's results and to the results of the ELISA and the SD Bioline RDT.

Just after reading, a photograph of each completed prototype RDT was taken using a mobile phone (Samsung Galaxy A8, with the RDT resting on a countertop and the phone held by hand in the usual way) and saved on a computer, and each RDT was also scanned using a scanner (CanoScan 9000F, resolution 300 dpi, Canon). Each RDT image (from both phone and scanner) was read by 2 additional blinded readers. Results were used to assess the potential for future quality assurance to be done using digital photographs.

Completed prototype RDTs were then stored at ambient temperature in a miniature house (which was constructed from wood in the hospital garden for a previous RDT stability study [14]), mimicking conditions in routine healthcare settings. After 7 days, the RDTs were read again using the same approach with 3 independent readers. During the 7 days, the temperature in the storage house was recorded every 30 minutes using an electronic thermometer (Tinytag Transit 2). Results were used to assess the potential for future quality assurance to be done using stored RDTs.

### Bioline RDT

To compare the prototype RDT and the Bioline RDT results under the same conditions, frozen samples with sufficient volume were also retested using the Bioline RDT. This testing followed the procedures described above: Laboratory technicians experienced in performing and reading RDTs performed the Bioline RDTs according to the manufacturer's instructions and loaded sera on the cassettes using a micropipette to ensure reproducibility of sample volume. Blinded reading was done as described above. The results were interpreted according to manufacturer's instructions: A red line appears at the NS1 test line level of the NS1 cassette in case of detection of DENV NS1. On antibody cassette, a red line appears at the IgM test line level in case of detection of anti-DENV IgM, and a red line at the IgG test line in case of detection of anti-DENV IgG. The results (negative or positive) are valid only if a red line appears at the control line level on both NS1 and antibody cassettes.

### ELISAs

Sera were stored at −20°C until enough samples accumulated to perform a full 96-well ELISA plate. Samples were tested by NS1 (Panbio Dengue Early ELISA, Abbott) and IgM (Panbio Dengue IgM Capture ELISA, Abbott), assays used for routine dengue diagnosis at Mahosot Hospital, following the manufacturer's instructions. Prototype RDT results were not made available to the technician performing the ELISAs. Equivocal ELISA results were counted as negative for final analysis.

### Observed RDT Use and Ease of Use Assessment

Five laboratory technicians who are involved in routine hospital practice and in performing RDTs for diagnosis were invited to participate in the study. The 5 technicians did not have prior specific training in using the dengue RDTs. The technicians were observed by a member of the study team (V.P.) while performing both the prototype and the Bioline RDTs on 4 serum samples each. For each RDT performed, the observer recorded on a form whether each step was performed correctly, performed incorrectly, or skipped. For each RDT performed, the technician and the observer each read and recorded the test result in a blinded fashion.

To assess how the participating technicians perceived the ease of use, after performing the RDTs each technician completed a questionnaire. The questionnaire asked about each step involved in performing and reading both RDTs, as well as the clarity of their instruction manuals. Technicians were also asked to compare the use of the prototype and Bioline RDTs with any other RDTs they had used previously.

### Sample Size Considerations

Because the sensitivity and specificity of the prototype RDTs compared to DENV qRT-PCR are not well established in the Greater Mekong region, it is recommended, based on statistical principles, to assume 50% sensitivity and specificity. This conservative estimate yields the largest required sample size, ensuring sufficient power to estimate sensitivity and specificity across all possible values, given a specific prevalence and confidence level. The prevalence of dengue using DENV qRT-PCR was approximately 40% from the in-house unpublished data. Using these parameters, estimating with 95% confidence and a 7.5% margin of error, a total of 427 samples would be required for estimating sensitivity. The sample size for estimating specificity using the same parameters gave a sample size of 285 samples. Thus, the sample size of 427 samples for sensitivity covers for estimation of both sensitivity and specificity. To account for indeterminate samples, we inflated the sample size by 20% and the required sample size was, therefore, 513 samples. During sample identification, there were 526 samples with valid data and we decided to include all the 526 samples, hence a gain in statistical precision for the estimates. The sample sizes were calculated in Stata MP version 18 using the following commands: “diagsampsi sensitivity 0.5, prev(0.4) width(0.075)” and “diagsampsi specificity 0.5, prev(0.4) width(0.075)” for estimating sensitivity and specificity, respectively.

### Data Management and Statistical Analysis

Data were entered in an Excel file and cleaned prior to statistical analysis. Statistical analysis was done using STATA software (version 14.0; StataCorp LLC), with 30% of the data records for the prototype RDT results checked by a member of the study team independent from the one who performed data entry (0.4% errors were detected and corrected). RDT results for each target (NS1, IgM, and IgG analyzed individually) were compared, and interreader variability was estimated by calculating percent agreement, as recommended by US Food and Drug Administration guidance [20], and the Cohen kappa coefficient was also calculated. Overall agreement was calculated as the ratio of number of concordant results (positive or negative) obtained by the 2 tests over the total number of samples tested. Positive and negative percent agreements were respectively calculated in the same way as for sensitivity and specificity. The term “percent agreements” is used when 2 tests or conditions are compared, with neither of them being standard. Sensitivity, specificity, and positive and negative predictive values, for the detection of NS1 or IgM alone or in combination (considered positive if at least 1 of the NS1 or IgM results was positive), were calculated using different reference standards—DENV qRT-PCR, DENV NS1 ELISA, and anti-DENV IgM ELISA—each individually and in combinations (qRT-PCR and NS1 ELISA combined, and all 3 tests combined). A sample was considered positive if any of the 2 or 3 combined tests yielded a positive result. A sample was considered negative if both or all 3 combined tests yielded a negative result. The ELISA results were interpreted according to the manufacturer's instructions. A DENV qRT-PCR result with a quantification cycle (Cq) ≤37 was considered positive. Indeterminate results from reference standard tests (equivocal results from ELISA, and Cq >37 for qRT-PCR) were counted as negative for the analysis. In case of an invalid RDT result, the RDT was repeated. Sample with missing data for 1 of the evaluated test or reference standard was excluded for the analysis. The 95% confidence intervals (CIs) for the sensitivity, specificity, and positive and negative predictive values were also calculated and reported.

### Ethics Approval

Patient sera and data used in this study were collected in the framework of the study titled “A prospective study of the causes of fever (UI-2 study) among patients admitted to Mahosot Hospital, Vientiane, Lao PDR.” All patients gave written informed consent for dengue investigation. Ethics approvals were provided by the Lao National Ethics Committee for Health Research (NECHR) numbers 2017.028NECHR and 2018.032NECHR, and the Oxford Tropical Research Ethics Committee (OxTREC006-07/2017).

## RESULTS

### Sample Inclusion

**Figure 1:**
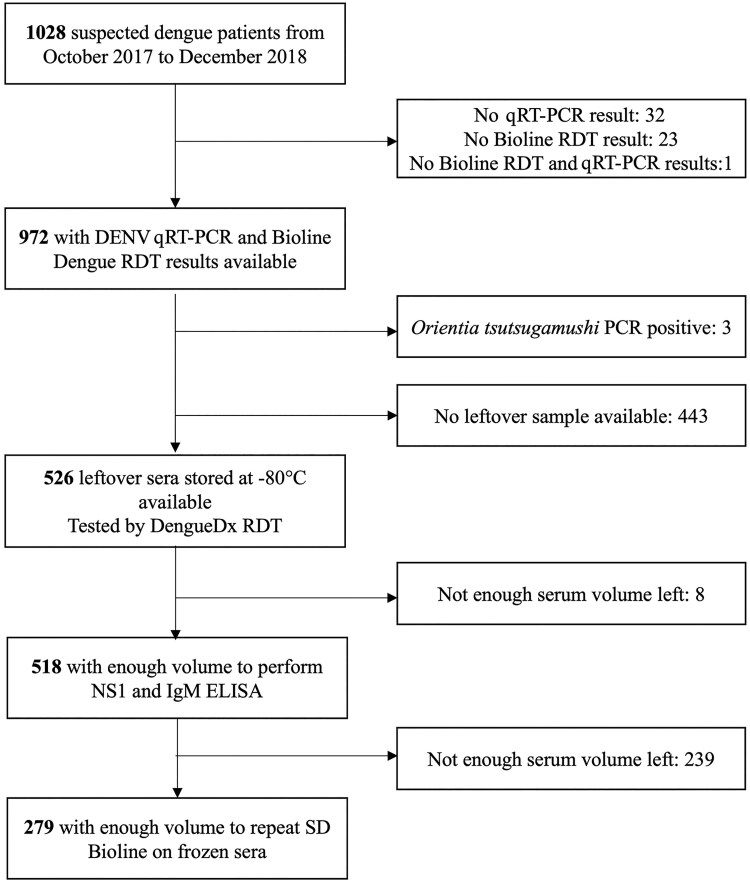
Flowchart for sample inclusion. Abbreviations: DENV, dengue virus; ELISA, enzyme-linked immunosorbent assay; IgM, immunoglobulin M; NS1, nonstructural protein 1; qRT-PCR, real-time reverse-transcription polymerase chain reaction; RDT, rapid diagnostic test.

Specimens from 1028 patients were submitted for dengue diagnosis from 1 October 2017 to 31 December 2018. For the current study, serum samples from 526 patients (Supplementary Table 1), stored at −80°C for 3–4 years, were available; 519 had enough volume to perform NS1 ELISA, and 522 for IgM ELISA. All results (RT-PCR, original Bioline RDT, prototype RDT, ELISA NS1, and ELISA IgM) were available for 518 samples. Of the 526 frozen samples, 279 had sufficient volume for retesting by the Bioline RDT (Figure 1).

### Prototype RDT Accuracy

#### Prototype and Bioline RDT Testing

Sixty percent of samples (326/526) tested positive by at least 1 of the dengue markers detected by the prototype RDT. The 2 readers’ results are displayed in Supplementary Table 2. Overall, 62 of 1578 (3.9%) RDT results were discordant between the 2 readers. Of the 526 frozen samples, 279 had enough volume for retesting by the Bioline RDT. Overall, 37 of 837 (4.4%) RDT results were discordant between the 2 independent readers (Supplementary Table 3). The results of the Bioline RDT from the initial testing (using fresh samples) and the retesting (using samples that had been stored at −80°C) showed 139 of 837 (16.6%) discordant results (Supplementary Table 4).

#### RDT Results Accuracy Compared to Reference Standards

**Figure 2.**
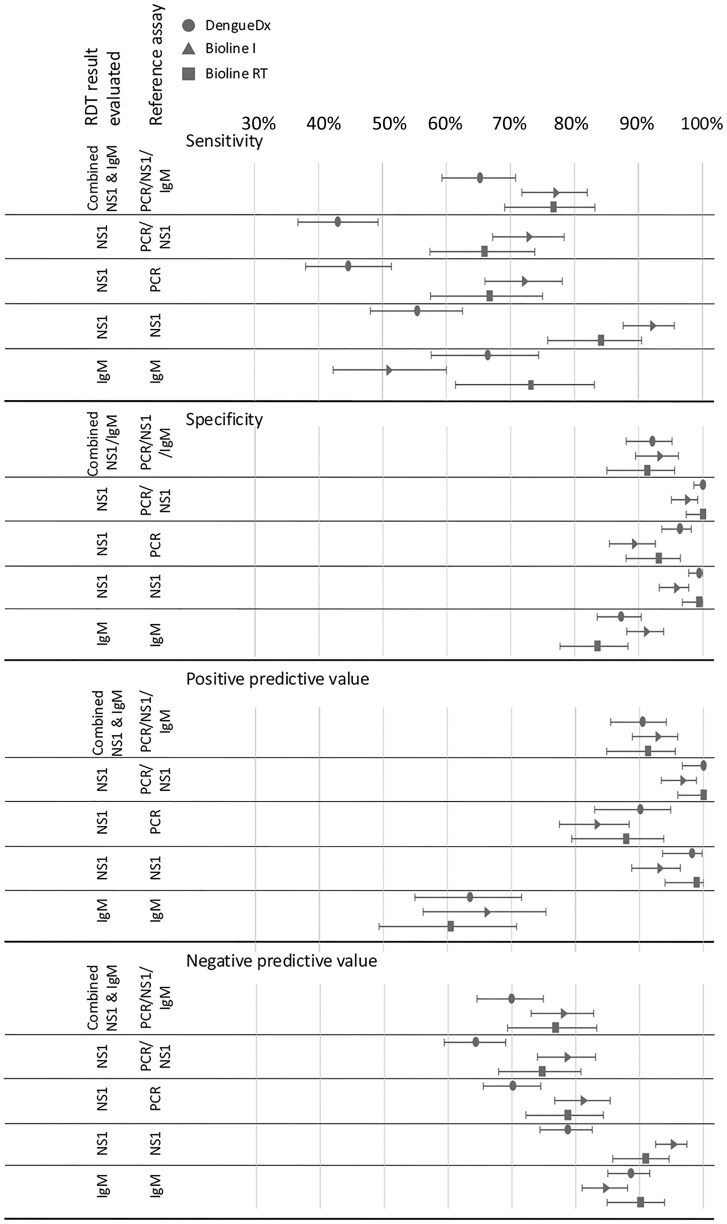
Accuracy of the prototype DengueDx and the SD Bioline RDTs performed on sera from hospitalized patients in Vientiane Capital, Lao People's Democratic Republic, calculated using qRT-PCR, NS1 ELISA, and IgM ELISA, alone or in combination, as reference. Results for DengueDx are displayed using circles. Bioline I: Initial results of the Bioline RDT done on fresh serum samples; results displayed using triangles. Bioline RT: results of the Bioline RDT retested on samples after storage at −80°C for 3–4 years; results displayed using squares. Combined NS1/IgM = RDT result was considered positive if at least 1 of the NS1 or IgM results was positive. PCR/NS1 = assay result was considered positive if at least 1 of the qRT-PCR or NS1 ELISA was positive. PCR/NS1/IgM = assay result was considered positive if at least 1 of the qRT-PCR, NS1 ELISA, or IgM ELISA was positive. Abbreviations: ELISA, enzyme-linked immunosorbent assay; IgM, immunoglobulin M; NS1, nonstructural protein 1; PCR, polymerase chain reaction; qRT-PCR, real-time reverse-transcription polymerase chain reaction; RDT, rapid diagnostic test.

Accuracy of the prototype and the Bioline RDTs for the detection of NS1 and/or IgM (evidence of acute infection) were estimated by comparing results with qRT-PCR, NS1 ELISA, and IgM ELISA alone or in combination as reference assays. Sensitivity, specificity, and positive and negative predictive values along with their 95% CIs are displayed in a forest plot (Figure 2, Supplementary Tables 5 and 6). The overall sensitivity of the prototype RDT (65.2% [95% CI, 59.3%–70.8%]) for the detection of acute dengue infection (one of RT-PCR, NS1 ELISA, or IgM ELISA positive) in stored samples was significantly lower than for the Bioline RDT (76.7% [95% CI, 69.1%–83.2%] on frozen samples, z-test *P* = .014; and 77.2% [95% CI, 71.8%–82.0%] on fresh samples, z-test *P* = .002). Even lower sensitivity was observed for the prototype RDT for NS1 detection alone: 43.0% (95% CI, 36.8%–49.3%) when using combined qRT-PCR and NS1 ELISA as reference, 44.6% (95% CI, 38.0%–51.4%) when using qRT-PCR as reference, and 55.4% (95% CI, 48.1%–62.5%) when using NS1 ELISA as reference. Sensitivity for detection of IgM was higher for the prototype RDT in stored samples (66.4% [95% CI, 57.6%–74.4%]) than for the Bioline RDT in fresh samples (51.1% [95% CI, 42.3%–60.0%]), but lower than the Bioline RDT in stored samples (73.2% [95% CI, 61.4%–83.1%]). The positive predictive value (PPV) of the prototype RDT for detection of NS1 was >90%.

### Prototype RDT Ease of Use

#### RDT Performance and Interpretation

**Figure 3.**
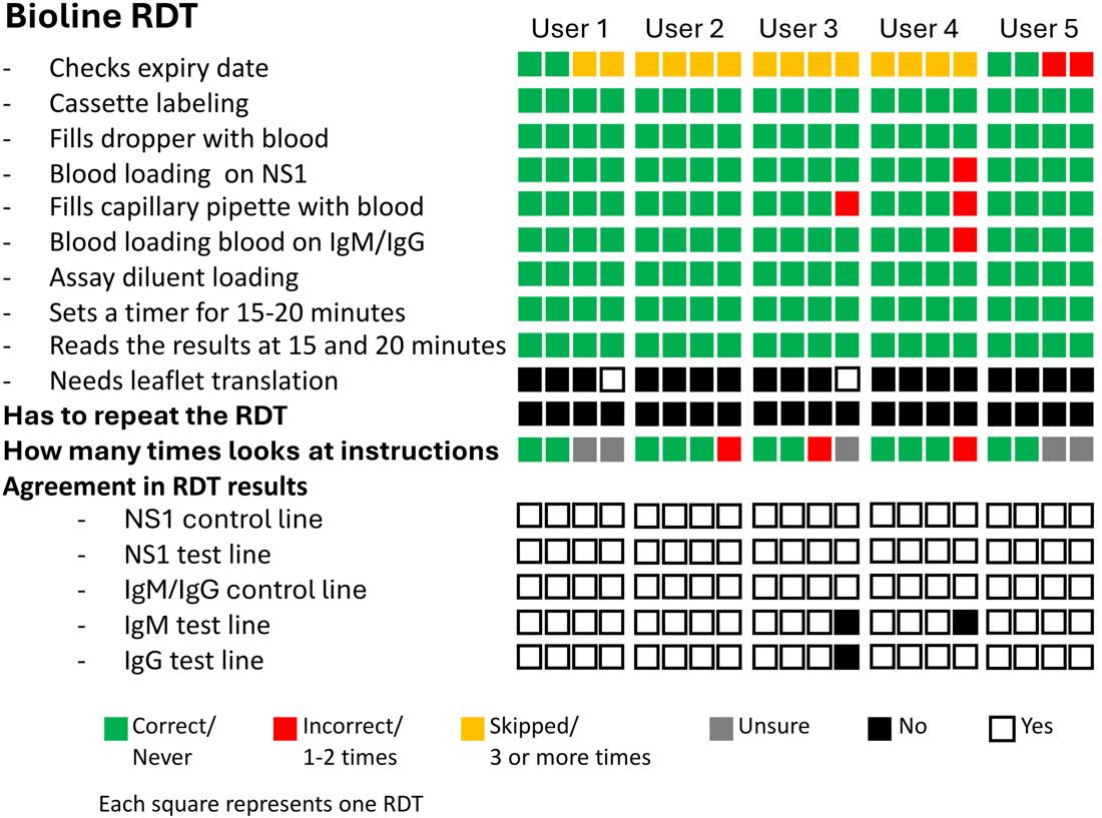
Results of the observation by a member of the study team of the 5 participants (laboratory technicians who were not specifically trained to use the dengue RDTs) when performing Bioline RDTs at Mahosot Hospital in Vientiane Capital, Lao People's Democratic Republic. Abbreviations: IgG, immunoglobulin G; IgM, immunoglobulin M; NS1, nonstructural protein 1; RDT, rapid diagnostic test.

**Figure 4.**
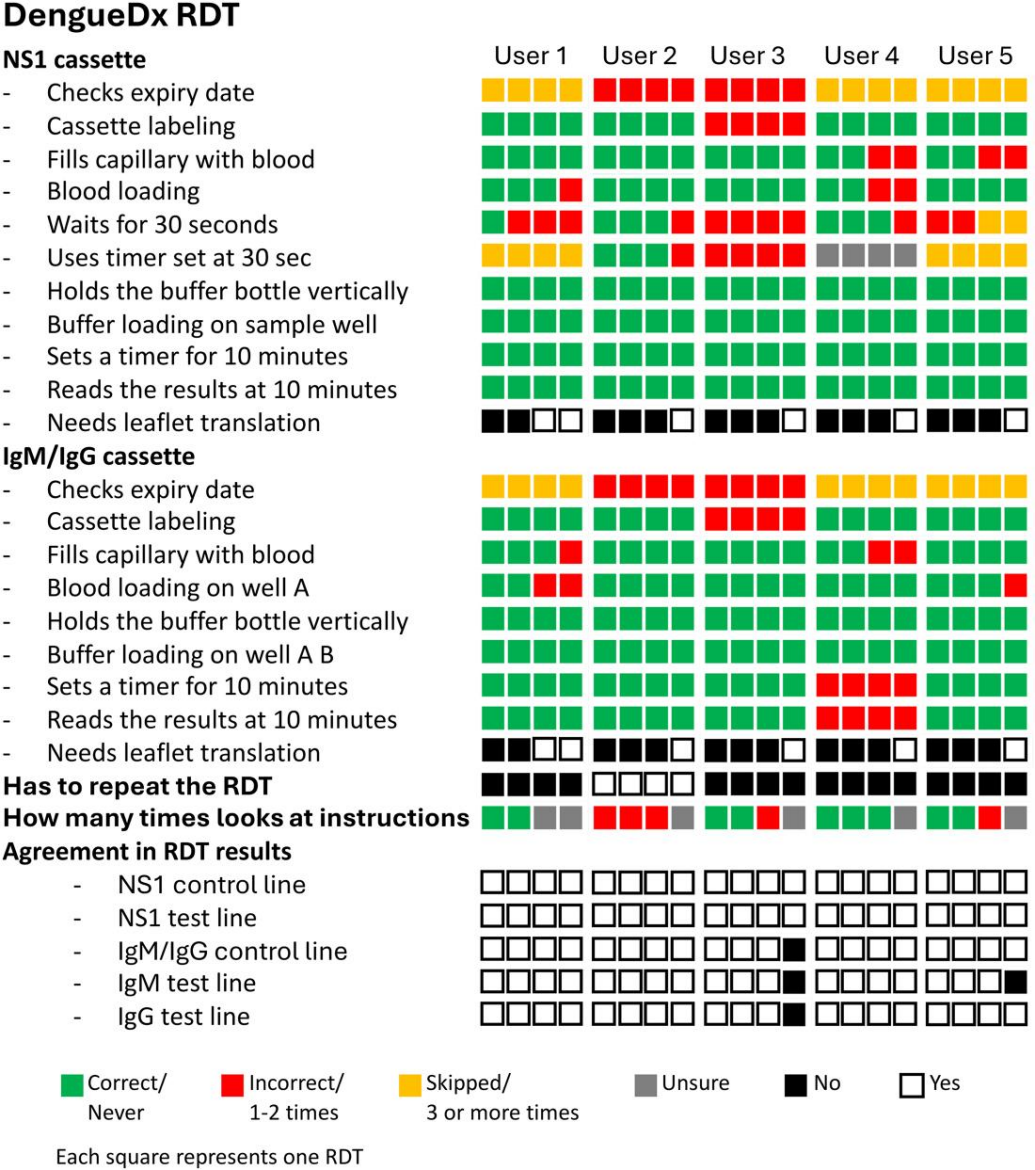
Results of the observation by a member of the study team of the 5 participants (laboratory technicians who were not specifically trained to use the dengue RDTs) when performing the prototype DengueDx RDTs at Mahosot Hospital in Vientiane Capital, Lao People's Democratic Republic. Abbreviations: IgG, immunoglobulin G; IgM, immunoglobulin M; NS1, nonstructural protein 1; RDT, rapid diagnostic test.

When participating technicians were observed performing the RDTs, they made errors in the steps of capillary pipette filling and sample loading more commonly for the prototype test (Figures 3 and 4) than for the Bioline test. Three participants did not wait for the required 30 seconds after sample loading before adding buffer to the prototype NS1 cassette.

Two participants required the manual to be explained in the Lao language by a study team member in order to perform the Bioline RDT, as did all 5 participants performing the prototype RDT. Additionally, 1 participant had to repeat all the prototype RDTs due to a misunderstanding of the instructions. For both prototype and Bioline RDTs, 2 participants disagreed with the observer on the IgM and/or IgG result for 1 of the 4 tests performed.

#### User Perceptions of Prototype RDT Ease of Use

**Figure 5.**
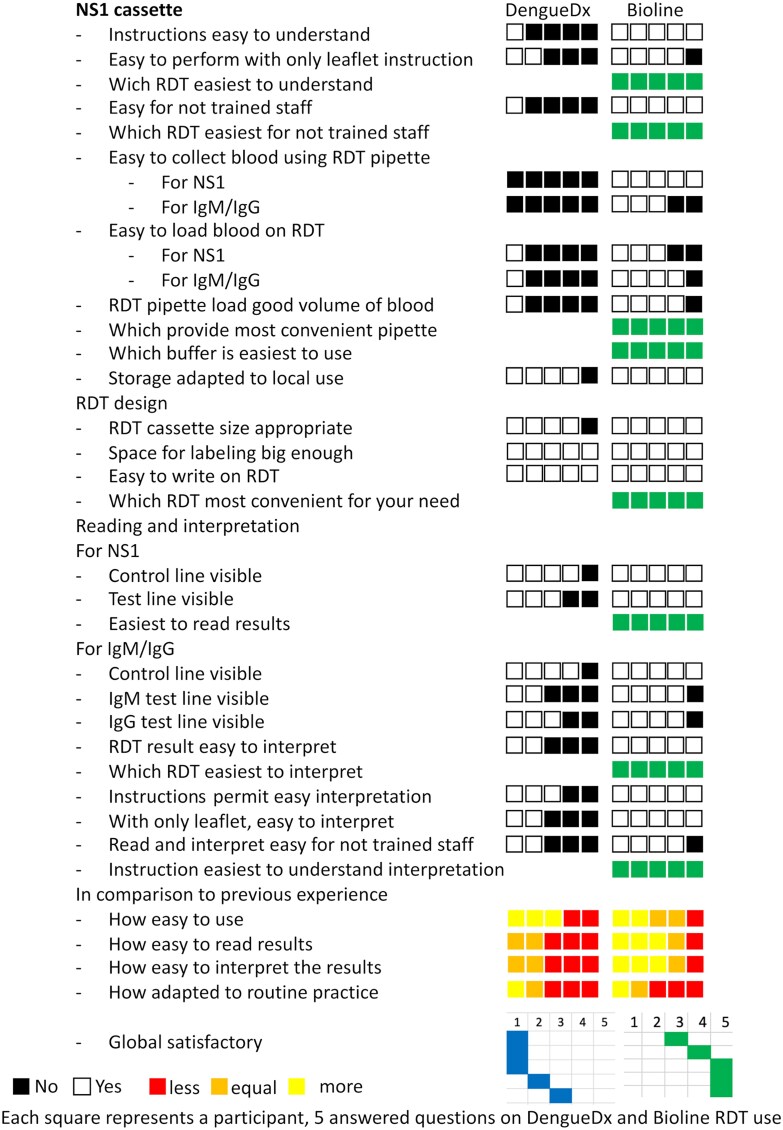
Results of the questionnaire completed by the 5 participants about the ease of use and interpretation when performing Bioline and prototype DengueDx RDTs at Mahosot Hospital in Vientiane Capital, Lao People's Democratic Republic. The participants were laboratory technicians who were experienced with routine clinical diagnostic laboratory work but who were not specifically trained to use the dengue RDTs. Abbreviations: IgG, immunoglobulin G; IgM, immunoglobulin M; NS1, nonstructural protein 1; RDT, rapid diagnostic test.

The participating technicians reported that the instructions provided on the package insert for the Bioline RDT were easier to understand than those for the prototype RDT, and that materials provided by the manufacturer for use with the Bioline RDT, such as plastic pipette, were easier to use and provided more accurate volume of serum and buffers than for prototype (Figure 5).

Participants did not report any preference concerning the device design, in terms of RDT size and surface space for labeling (numbers, name, date of test, etc), which was satisfactory for both RDTs. In a comparison of the control and test lines, visibility was satisfactory for all participants for NS1 for the Bioline RDT, but not satisfactory for 2 participants on the prototype RDT. For the IgM/IgG cassette, visibility was not satisfactory for 1 participant for the Bioline RDT and 3 participants for prototype.

Three of the 5 participants felt that the results from the prototype RDT were not easy to interpret. All participants felt that the instructions in the package insert, on how to interpret the test results, were easier to understand for the SD Bioline RDT than for the prototype RDT. Three of the 5 participants felt that the instructions provided in the prototype package alone did not permit an easy interpretation of the test results (Figure 5).

### Remote RDT Result Interpretation

#### Interpretation of Prototype RDT Results From Scans and Photographs

The image-based results were compared to the results directly read from the RDTs (Table 1). The negative percent agreements were >99% for both image tools. However, moderate to low positive percent agreements were observed, especially for photographs with 61.3% (95% CI, 51.5%–70.4%), 29.7% (95% CI, 22.2%–38.1%), and 55.0% (95% CI, 48.7%–61.2%), for NS1, IgM, and IgG detection, respectively. This suggests that some positive test bands, perhaps those that were weaker or fainter, could not be detected from images.

**Table 1.** Comparison of Prototype DengueDx Rapid Diagnostic Test (RDT) Results Read Directly From the RDT and Read From Scanned Pictures or Photographs (Taken Using Mobile Phone), and When Read 7 Days After Performing the RDT, at Mahosot Hospital in Vientiane Capital, Lao People’s Democratic Republic

Agreement	RDT vs Scan Image–Based Results			RDT vs Photograph Image–Based Results			D0 vs D7 Reading		
	NS1	IgM	IgG	NS1	IgM	IgG	NS1	IgM	IgG
Overall percent agreement^a^	98.3% (96.8–99.2)	89.7% (86.8–92.2)	90.7% (87.9–93.0)	91.8% (89.1–94.0)	81.6% (78.0–84.8)	77.8% (74.0–81.2)	91.6% (88.9–93.9)	68.4% (64.3–72.4)	85.7% (82.5–88.6)
Positive percent agreement^b^	94.6% (88.6–98.0)	61.6% (52.9–69.7)	81.1% (75.9–85.7)	61.3% (51.5–70.4)	29.7% (22.2–38.1)	55.0% (48.7–61.2)	98.2% (93.6–99.8)	98.6% (94.9–99.8)	99.6% (97.9–100)
Negative percent agreement^c^	99.3% (97.9–99.9)	99.7% (98.6–100)	100% (98.6–100)	100% (99.1–100)	100% (99.1–100)	100% (98.6–100)	89.9% (86.6–92.6)	57.7% (52.6–62.7)	72.2% (66.4–77.5)

Values in brackets are the 95% confidence interval.

Abbreviations: D0, day 0 (day of performing the rapid diagnostic test); D7, day 7 (7 days after performing the rapid diagnostic test); IgG, immunoglobulin G, IgM, immunoglobulin M; NS, nonstructural protein 1; RDT, rapid diagnostic test.

^a^Overall percent agreement is the ratio of the concordant results (positive or negative) over the total number of samples tested).

^b^Positive percent agreement is the ratio of the concordant positive results over the total number of samples found positive when reading results directly from RDT (RDT vs scan image–based results, RDT vs photograph image–based results), or when reading on day 0 (D0 vs D7).

^c^Negative percent agreement is the ratio of the concordant negative results over the total number of samples found negative when reading results directly from RDT (RDT vs scan, RDT vs photograph) or when reading on day 0 (D0 vs D7).

#### Interpretation of Prototype RDT Results After 7 Days at Ambient Temperature

**Figure 6.**
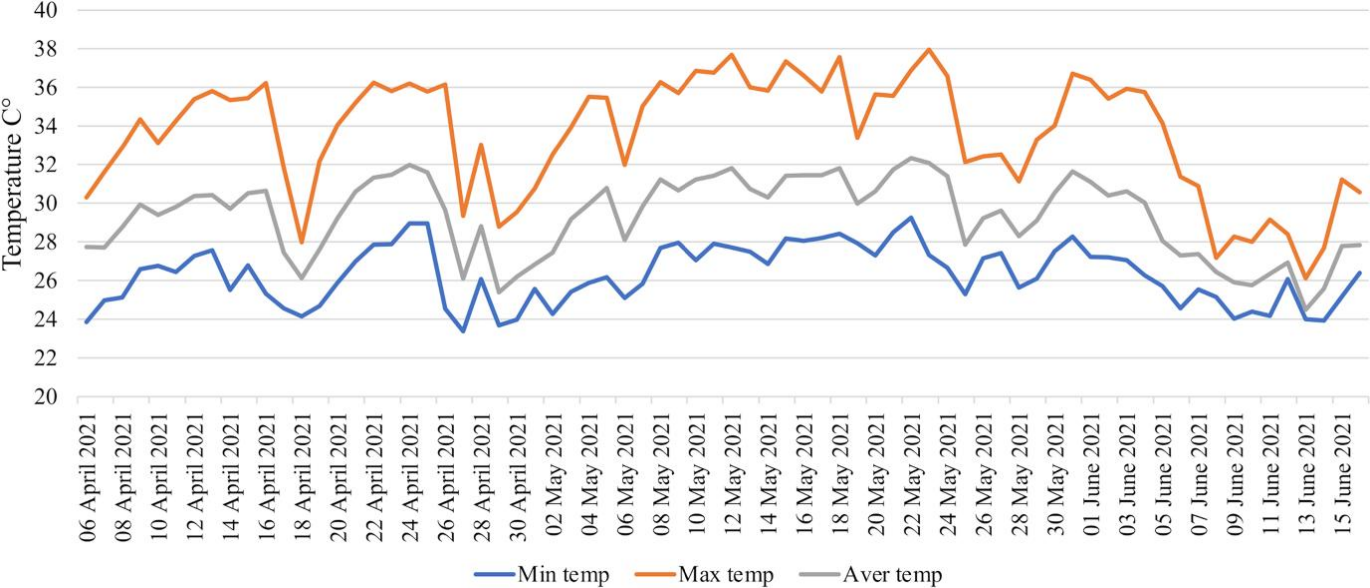
Daily temperature recorded in a miniature purpose-built wooden house over the 3-month period of the rapid diagnostic test storage study, in Vientiane Capital, Lao People's Democratic Republic. Abbreviations: Aver temp: daily average temperature; Max temp: daily maximum temperature; Min temp: daily minimum temperature.

After 7 days’ storage in the miniature house, the prototype RDTs were read again by 2 readers who were blinded to the results read at day 0. Average daily temperatures recorded in the house over the period of RDT storage ranged from 24.5°C to 32.3°C (Figure 6); the lowest temperature recorded was 23.4°C and the highest was 38.0°C. Results read at day 7 were compared to the results read at day 0 (Table 1). Positive percent agreements were >98%, while negative percent agreements were lower, especially for IgM (57.7% [95% CI, 52.6%–62.7%]).

## DISCUSSION

In this study, the sensitivities for NS1 antigen detection of the prototype RDT were 43.0%, 44.6%, and 55.4% when compared with combined qRT-PCR and NS1 ELISA, qRT-PCR, or NS1 ELISA as reference assays, respectively. The sensitivities for NS1 Bioline RDT were 65.9%, 66.7%, and 84.1% compared with combined qRT-PCR and NS1 ELISA, qRT-PCR, or NS1 ELISA as reference assays, respectively, significantly higher than for the prototype RDT. Haider et al did a systematic review of the literature reporting data on the performance of dengue RDTs that detect NS1 and/or IgM to diagnose DENV infection [9]. When analyzing NS1 detection performance in acute phase, the RDT sensitivities ranged from 41.8% to 93.1% and the cumulative sensitivity was 71% (standard deviation, 14.7%). Sensitivity was >60% in 84% [20] of the 25 RDTs evaluated. The NS1 antigen detection sensitivities reported for other commercial RDTs therefore are higher than that in our evaluation. Low sensitivity would explain the interreader discordance that we observed in our study for reading NS1 RDT results. Indeed, when we looked at the scanned photos of the NS1 RDTs with discordant results, all showed weak or no visible band on photos. However, the PPV of the prototype RDT for the detection of DENV infection (positive by any of RT-PCR, NS1 ELISA, or IgM ELISA) was >90%. Therefore, in a context of high DENV circulation during an outbreak, the use of the prototype RDT would help with quick identification of DENV infection. The performance of IgM detection is more variable from one RDT brand to another, with sensitivity ranging from 11.7% to 89.9% and a cumulative sensitivity of 40.32% (standard deviation, 26.02%) [9]. The prototype RDT would perform satisfactorily for the detection of IgM, in comparison to other commercial RDTs, with a sensitivity of 66.4% observed in our study.

During the ease of use assessment, we observed that the Bioline RDT performed better than the prototype RDT, with minor mistakes done at different steps and 1 of the 5 participants misunderstanding the instructions and having to repeat RDT testing. However, the ability of the participants to interpret the RDT results was similar for both brands. The overall satisfaction of the participants was much better for Bioline than for the prototype RDT. The instructions provided on the RDT leaflet were found to be more difficult to understand for prototype than for Bioline. Inconsistency between the narrative and the picture were noticed on the prototype leaflet. At the time of the study, the prototype RDT was not commercialized, so the leaflet used for the study was a pilot one; eventually the final version of the leaflet, with the inconsistency modified, would be expected to be as easy as the Bioline one to understand. For the prototype RDT, the NS1 and IgM/IgM strips are in 2 separate cassettes, and the test targets (dengue NS1 or dengue IgM/IgG) are not indicated on the cassettes. These were reported by the participants as important concerns for the use of the prototype RDT in routine diagnosis. The dengue Bioline RDT has been widely used in the Microbiology Laboratory at Mahosot Hospital for various studies and diagnostic activities, and therefore all staff involved in diagnostic testing had already used the dengue Bioline RDT before the current study. This could have biased their judgment when comparing the ease of use of both RDTs. The fact that they were familiar with the Bioline RDT could have played a significant role in the perception they had that Bioline was easier to use than the prototype. However, this does not invalidate the perception results and highlights the fact that it could be even more difficult to implement a new test in a place already familiar with similar practice. Therefore, local practice, absence of knowledge about a test, or ingrained habits of using another test must be taken into consideration when developing and implementing new tests.

Ninety-five percent of the positive results read directly from RDTs were also found positive when reading results from scanned photographs. Lower agreement was observed for IgM (62%) and IgG (81%) results. Whereas image-based results from scanned RDTs could be satisfactory, at least for NS1, this was not the case when reading results from photographs taken with the mobile phone. Only 61% of the direct NS1 positive results were found positive when reading results from the photographs, and only 30% for IgM and 55% for IgG. Weak bands were more difficult to read from a picture than directly from the RDT. Therefore, weaker bands obtained for IgM and IgG, in comparison to NS1 bands, would explain the lower correlation of IgM and IgG readings on scans and mobile phone photographs. Mobile phones with lower resolution cameras than a scanner will have more difficulty to display the weakest bands. The use of smartphones is widespread in low-income countries, so this would appear to be the tool of choice to help with reporting of results from remote areas. However, our findings show that taking digital photographs of RDTs with a smartphone or tablet does not permit accurate reporting of RDT results.

Another approach would be to collect RDTs from the field, then read the results in a central laboratory. In our study, we simulated field conditions by leaving completed RDTs in a miniature house for 7 days. The RDTs were exposed to an average daily temperature between 24.5°C and 32.3°C. High correlation (>98%) for positive results was obtained between the reading at day 0 and the reading at day 7. However, lower correlations were observed for negative results (89.9% for NS1, 57.7% for IgM, and 72.2% for IgG). This is because in an RDT that originally correctly showed a negative result, a test band can develop with the passage of time, leading to a false-positive result when the RDT is reread after a period of storage. However, combining reading RDT results from photographs, and delayed reading of RDT collected from the field, would allow an assessment of the overall quality of the performance of RDTs in the field.

The main limitations of our study were that the RDT evaluation was done on frozen samples and that all frozen samples could not be retested by Bioline RDT. We were not able to analyze the results according to DENV serotype, and clinical data were not available to estimate any differences of RDT performance according to clinical presentation.

## CONCLUSIONS

With PPV >90% for NS1 detection, the prototype RDT would be useful for helping with quick identification of dengue infections in acute phases during an outbreak. Improvement in test sensitivity, as well as in device design (single antigen/antibody device, adapted pipette), would make this RDT better adapted to the Lao and similar contexts, while aligning more closely to the ASSURED criteria. Further evaluations would be necessary to determine the performance of the test according to primary/secondary infection status, infecting serotype, and severity. Prospective evaluation of any new RDT during an outbreak, in the field, or in different healthcare settings, would enable a more accurate estimation of the added value of the use of the RDT for dengue surveillance in remote areas, which is essential to fulfil the ASSURED criteria. We shared results of this evaluation with the test manufacturers, who informed us that work on reagent optimization is under way (personal communication, Global Access Diagnostics August 2024).

Currently no RDTs for dengue are prequalified by the World Health Organization, making it difficult for countries selecting tests to make procurement decisions. A target product profile for tests to perform prevaccination screening for dengue serostatus has been proposed [21], but there is no target product profile for tests to diagnose current dengue infection. Good quality, affordable point-of-care tests with high sensitivity and specificity for diagnosis of dengue are urgently needed.

## Supplementary Material

ofag422_Supplementary_Data

## Supplementary Data

Supplementary materials are available at *Open Forum Infectious Diseases* online. Consisting of data provided by the authors to benefit the reader, the posted materials are not copyedited and are the sole responsibility of the authors, so questions or comments should be addressed to the corresponding author.

## References

[1] World Health Organization . Dengue haemorrhagic fever: diagnosis, treatment, prevention and control. 1997. Available at: https://apps.who.int/iris/handle/10665/41988. Accessed July 2026.

[2] Bhatt S, Gething PW, Brady OJ, et al The global distribution and burden of dengue. Nature 2013; 496:504–7. 23563266 10.1038/nature12060 PMC3651993

[3] World Health Organization, Regional Office for South-East Asia . Dengue case management: report of an informal expert consultation, Colombo, Sri Lanka, 12–14 August 2013. New Delhi: WHO Regional Office for South-East Asia; **2014**. Available at: https://iris.who.int/bitstream/handle/10665/204949/B5060.pdf?sequence=1. Accessed July 2026.

[4] Khampapongpane B, Lewis HC, Ketmayoon P, et al National dengue surveillance in the Lao People's Democratic Republic, 2006-2012: epidemiological and laboratory findings. Western Pac Surveill Response J 2014; 5:7–13. 10.5365/WPSAR.2014.5.1.001 PMC3984965 24734212

[5] Conlan JV, Vongxay K, Khamlome B, et al Patterns of flavivirus seroprevalence in the human population of northern Laos. Am J Trop Med Hyg 2015; 93:1010–3. 26304925 10.4269/ajtmh.15-0072 PMC4703266

[6] Mayxay M, Castonguay-Vanier J, Chansamouth V, et al Causes of non-malarial fever in Laos: a prospective study. Lancet Glob Health 2013; 1:e46–54. 24748368 10.1016/S2214-109X(13)70008-1 PMC3986032

[7] Dubot-Pérès A, Vongphrachanh P, Denny J, et al An epidemic of dengue-1 in a remote village in rural Laos. PLoS Negl Trop Dis 2013; 7:e2360. 23951379 10.1371/journal.pntd.0002360 PMC3738459

[8] Castonguay-Vanier J, Klitting R, Sengvilaipaseuth O, et al Molecular epidemiology of dengue viruses in three provinces of Lao PDR, 2006-2010. PLoS Negl Trop Dis 2018; 12:e0006203. 29377886 10.1371/journal.pntd.0006203 PMC5805359

[9] Haider M, Yousaf S, Zaib A, Sarfraz A, Sarfraz Z, Cherrez-Ojeda I. Diagnostic accuracy of Various immunochromatographic tests for NS1 antigen and IgM antibodies detection in acute dengue virus infection. Int J Environ Res Public Health 2022; 19:8756. 35886607 10.3390/ijerph19148756 PMC9324781

[10] Chong ZL, Sekaran SD, Soe HJ, Peramalah D, Rampal S, Ng CW. Diagnostic accuracy and utility of three dengue diagnostic tests for the diagnosis of acute dengue infection in Malaysia. BMC Infect Dis 2020; 20:210. 32164538 10.1186/s12879-020-4911-5 PMC7069157

[11] Lee H, Ryu JH, Park HS, et al Comparison of six commercial diagnostic tests for the detection of dengue virus non-structural-1 antigen and IgM/IgG antibodies. Ann Lab Med 2019; 39:566–71. 31240885 10.3343/alm.2019.39.6.566 PMC6660329

[12] Yow KS, Aik J, Tan EYM, Ng LC, Lai YL. Rapid diagnostic tests for the detection of recent dengue infections: an evaluation of six kits on clinical specimens. PLoS One 2021; 16:e0249602. 33793682 10.1371/journal.pone.0249602 PMC8016316

[13] Blacksell SD, Jarman RG, Bailey MS, et al Evaluation of six commercial point-of-care tests for diagnosis of acute dengue infections: the need for combining NS1 antigen and IgM/IgG antibody detection to achieve acceptable levels of accuracy. Clin Vaccine Immunol 2011; 18:2095–101. 22012979 10.1128/CVI.05285-11 PMC3232692

[14] Phommasone K, Sengvilaipaseuth O, de Lamballerie X, et al Temperature and the field stability of a dengue rapid diagnostic test in the tropics. Am J Trop Med Hyg 2015; 93:33–9. 25962773 10.4269/ajtmh.15-0142 PMC4497900

[15] Sengvilaipaseuth O, Phommasone K, de Lamballerie X, et al Temperature of a dengue rapid diagnostic test under tropical climatic conditions: a follow up study. PLoS One 2017; 12:e0170359. 28129346 10.1371/journal.pone.0170359 PMC5271306

[16] Global Access Diagnostics . Mologic to become a ‘Social Enterprise’ after acquisition by social impact funders and investors. 2026. Available at: https://www.globalaccessdx.com/mologic-to-become-a-social-enterprise-after-acquisition-by-social-impact-funders-and-investors/. Accessed July 2026.

[17] Zeltins A . Construction and characterization of virus-like particles: a review. Mol Biotechnol 2013; 53:92–107. 23001867 10.1007/s12033-012-9598-4 PMC7090963

[18] Bossuyt PM, Reitsma JB, Bruns DE, et al STARD 2015: an updated list of essential items for reporting diagnostic accuracy studies. Radiology 2015; 277:826–32. 26509226 10.1148/radiol.2015151516

[19] Leparc-Goffart I, Baragatti M, Temmam S, et al Development and validation of real-time one-step reverse transcription-PCR for the detection and typing of dengue viruses. J Clin Virol 2009; 45:61–6. 19345140 10.1016/j.jcv.2009.02.010

[20] US Food and Drug Administration . Statistical guidance of reporting results from studies evaluating diagnostic tests. **2007**. Available at: https://www.hhs.gov/guidance/sites/default/files/hhs-guidance-documents/FDA/Guidance-for-Industry-and-FDA-Staff---Statistical-Guidance-on-Reporting-Results-from-Studies-Evaluating-Diagnostic-Tests-%28PDF-Version%29.pdf. Accessed July 2026.

[21] Fongwen N, Wilder-Smith A, Gubler DJ, et al Target product profile for a dengue pre-vaccination screening test. PLoS Negl Trop Dis 2021; 15:e0009557. 34324505 10.1371/journal.pntd.0009557 PMC8320982

