# Supplementary material for: Accuracy and Usability of a New Rapid Diagnostic Test for the Diagnosis of Dengue in Vientiane, Laos
Source: Open Forum Infect Dis. 2026 Jul 14;13(7):ofag422. doi: 10.1093/ofid/ofag422 (PMC13367443; doi:10.1093/ofid/ofag422)
Supplement: ofag422_Supplementary_Data [file ofag422_supplementary_data.docx]

**Table S1**: Characteristics of patients who provided samples for an evaluation of dengue diagnostic tests in Vientiane, Laos.

|  | N=521* |
| --- | --- |
| Age (year), median (IQR) | 25 (16-37) |
| Age group:  Children (<15 year old)  Adult (≥15 year old) | 115 (22.2)  404 (77.8) |
| Gender  Female, n (%)  Male, n (%) | 264 (50.7)  257 (49.3) |
| Ward:  Paediatrics  ICU Adult  Infectious Disease Adult  International clinic  Tuberculosis  Other | 116 (22.3)  13 (2.5)  346 (66.5)  19 (3.7)  11 (2.2)  16 (3.1) |
| Number of days of illness, median (IQR) | 4 (3-5) |

* data available for 521 of the 526 included patients.

**Table S2**. Comparison of prototype DengueDx dengue rapid diagnostic test (RDT) results read independently by two laboratory technicians at Mahosot Hospital in Vientiane, Lao PDR

| **NS1 results** | | | **Reader 2** | | **Total** |
| --- | --- | --- | --- | --- | --- |
|  |  |  | **Positive** | **Negative** |  |
| **Reader 1** | **Positive** | | 108 | 3 | 111 |
|  | **Negative** | | 10 | 405 | 415 |
| **Total** | | | 118 | 408 | 526 |
| Overall percent agreement (95% CI): 97.5% (95.8-98.7)  Positive percent agreement (95% CI): 91.5% (85.0-95.9)  Negative percent agreement (95% CI): 99.3% (97.9-99.8)  Cohen’s kappa coefficient (95% CI): 0.93 (0.89-0.97) | | | | | |
| **IgM results** | | | | | |
| **Reader 1** | | **Positive** | 133 | 14 | 147 |
|  |  | **Negative** | 7 | 372 | 379 |
| **Total** | | | 140 | 386 | 526 |
| Overall percent agreement (95%CI): 96.0% (94.0-97.5)  Positive percent agreement (95%CI): 95.0% (90.0-98.0)  Negative percent agreement (95%CI): 96.4% (94.0-98.0)  Cohen’s kappa coefficient (95%CI): 0.90 (0.86-0.94) | | | | | |
| **IgG results** | | | | | |
| **Reader 1** | | **Positive** | 242 | 16 | 258 |
|  |  | **Negative** | 12 | 256 | 268 |
| **Total** | | | 254 | 272 | 526 |
| Overall percent agreement (95%CI): 94.7% (92.4-96.4)  Positive percent agreement (95%CI): 95.3% (91.9-97.5)  Negative percent agreement (95%CI): 94.1% (90.6-96.6)  Cohen’s kappa coefficient: 0.89 (0.86-0.93) | | | | | |

NS: nonstructural protein, IgM: immunoglobulin M, IgG : immunoglobulin G, CI : confidence interval.

**Table S3**. Comparison of Bioline dengue rapid diagnostic test (RDT) results, performed in frozen sera, read independently by two laboratory technicians at Mahosot Hospital in Vientiane, Lao PDR

| **NS1 results** | | | **Reader 2** | | **Total** |
| --- | --- | --- | --- | --- | --- |
|  |  |  | **Positive** | **Negative** |  |
| **Reader 1** | **Positive** | | 89 | 4 | 93 |
|  | **Negative** | | 3 | 183 | 186 |
| **Total** | | | 92 | 187 | 279 |
| Overall percent agreement (95% CI): 97.6% (95.2 – 99.0)  Positive percent agreement (95% CI): 96.7% (90.8 – 99.3)  Negative percent agreement (95% CI): 97.9% (94.6 – 99.4)  Cohen’s kappa coefficient (95% CI): 0.94 (0.90-0.99) | | | | | |
| **IgM results** | | | | | |
| **Reader 1** | | **Positive** | 78 | 6 | 84 |
|  |  | **Negative** | 6 | 189 | 195 |
| **Total** | | | 84 | 195 | 279 |
| Overall percent agreement (95% CI): 95.7% (92.6 – 97.8)  Positive percent agreement (95% CI): 92.9% (85.1- 97.3)  Negative percent agreement (95% CI): 96.9% (93.4 – 98.9)  Cohen’s kappa coefficient (95% CI): 0.90 (0.84-0.95) | | | | | |
| **IgG results** | | | | | |
| **Reader 1** | | **Positive** | 163 | 6 | 169 |
|  |  | **Negative** | 12 | 98 | 110 |
| **Total** | | | 175 | 104 | 279 |
| Overall percent agreement (95% CI): 93.5% (90.0 – 96.1)  Positive percent agreement (95% CI): 93.1% (88.3 – 96.4)  Negative percent agreement (95% CI): 94.2% (87.9 – 97.9)  Cohen’s kappa coefficient (95% CI): 0.86 (0.80-0.92) | | | | | |

NS: nonstructural protein, IgM: immunoglobulin M, IgG : immunoglobulin G, CI : confidence interval.

**Table S4.** Comparison of Bioline dengue RDT results performed initially on fresh samples and retested on samples after storage at -80ºC for three to four years at Mahosot Hospital in Vientiane, Lao PDR

| **NS1 results** | | | **Bioline initial** | | **Total** |
| --- | --- | --- | --- | --- | --- |
|  |  |  | **Positive** | **Negative** |  |
| **Bioline retesting** | **Positive** | | 89 | 2 | 91 |
|  | **Negative** | | 15 | 173 | 188 |
| **Total** | | | 104 | 175 | 279 |
| Overall percent agreement (95% CI): 93.9% (90.4 – 96.4)  Positive percent agreement (95% CI): 85.6% (77.3 – 91.7)  Negative percent agreement (95% CI): 98.9% (95.9 – 99.9)  Cohen’s kappa coefficient (95% CI): 0.87 (0.81-0.93) | | | | | |
| **IgM results** | | | | | |
| **Bioline retesting** | | **Positive** | 43 | 43 | 86 |
|  |  | **Negative** | 5 | 188 | 193 |
| **Total** | | | 48 | 231 | 279 |
| Overall percent agreement (95% CI):82.8% (77.8 – 87.0)  Positive percent agreement (95% CI): 89.6% (77.3 – 96.5)  Negative percent agreement (95% CI): 81.4% (75.8 – 86.2)  Cohen’s kappa coefficient (95% CI): 0.54 (0.43-0.65) | | | | | |
| **IgG results** | | | | | |
| **Bioline retesting** | | **Positive** | 108 | 70 | 178 |
|  |  | **Negative** | 4 | 97 | 101 |
| **Total** | | | 112 | 167 | 279 |
| Overall percent agreement (95% CI): 73.4% (67.8 – 78.5)  Positive percent agreement (95% CI): 96.4% (91.1 – 99.0)  Negative percent agreement (95% CI): 58.1% (50.2 – 65.7)  Cohen’s kappa coefficient (95% CI): 0.50 (0.41-0.59) | | | | | |

NS: nonstructural protein, IgM: immunoglobulin M, IgG : immunoglobulin G, CI : confidence interval.

**Table S5.** Accuracy of the prototype DengueDx and SD Bioline RDTs performed on sera from hospitalized patients in Vientiane Capital, Lao PDR, calculated using qRT-PCR, NS1 and IgM ELISA, alone or in combination, as reference.

| **Results used from RDT** | | **NS1 or IgM*** | **NS1** | | | **IgM** |
| --- | --- | --- | --- | --- | --- | --- |
| **Reference assay** | | **qRT-PCR/NS1/IgM ELISA**** | **qRT-PCR/NS1 ELISA°** | **qRT-PCR^$^** | **NS1 ELISA^€^** | **IgM ELISA^€^** |
| **DengueDx** **RDT (frozen samples)** | **Sensitivity, % (95%CI)** | 65.2 (59.3 – 70.8) | 43.0 (36.8 – 49.3) | 44.6 (38.0 – 51.4) | 55.4 (48.1 – 62.5) | 66.4 (57.6 – 74.4) |
|  | **Specificity, % (95%CI)** | 92.1 (88.0 – 95.2) | 100 (98.6 – 100) | 96.4 (93.6 – 98.2) | 99.4 (97.8 – 100) | 87.2 (83.5 – 90.4) |
|  | **PPV, % (95%CI)** | 90.5 (85.5 – 94.2) | 100 (96.7 – 100) | 90.1 (83.0 – 94.9) | 98.2 (93.6 – 99.8) | 63.5 (54.9 – 71.6) |
|  | **NPV, % (95%CI)** | 69.9 (64.5 – 74.9) | 64.3 (59.4 – 69.0) | 70.1 (65.5 – 74.5) | 78.7 (74.4 – 82.6) | 88.6 (85.0 – 91.6) |
| **Bioline RDT initial (fresh samples)** | **Sensitivity, % (95%CI)** | 77.2 (71.8 – 82.0) | 73.0 (67.2 – 78.4) | 72.3 (66.0 – 78.1) | 92.3 (87.6 – 95.6) | 51.1 (42.3 – 60.0) |
|  | **Specificity, % (95%CI)** | 93.4 (89.5 – 96.2) | 97.7 (95.1 – 99.2) | 89.4 (85.4 – 92.6) | 96.0 (93.2 – 97.8) | 91.3 (88.1 – 93.9) |
|  | **PPV, % (95%CI)** | 93.0 (88.9 – 96.0) | 96.9 (93.4 – 98.9) | 83.5 (77.5 – 88.4) | 93.3 (88.8 – 96.4) | 66.3 (56.2 – 75.4) |
|  | **NPV, % (95%CI)** | 78.2 (73.0 – 82.8) | 78.8 (74.0 – 83.1) | 81.3 (76.7 – 85.4) | 95.4 (92.5 – 97.4) | 84.8 (81.0 – 88.1) |
| **Bioline RDT retesting (frozen samples)** | **Sensitivity, % (95%CI)** | 76.7 (69.1 –83.2) | 65.9 (57.4 – 73.8) | 66.7 (57.5 – 75.0) | 84.1 (75.8 – 90.5) | 73.2 (61.4 – 83.1) |
|  | **Specificity, % (95%CI)** | 91.3 (85.0 – 95.6) | 100 (97.4 – 100) | 93.1 (88.0 – 96.5) | 99.4 (96.8 – 100) | 83.5 (77.7 – 88.3) |
|  | **PPV, % (95%CI)** | 91.3 (84.9 – 95.6) | 100 (96.0 – 100) | 87.9 (79.4 – 93.8) | 98.9 (94.0 – 100) | 60.5 (49.3 –70.8) |
|  | **NPV, % (95%CI)** | 76.8 69.3 – 83.3) | 74.7 (67.9 – 80.8) | 78.7 (72.2 – 84.3) | 90.9 (85.8 – 94.6) | 90.1 (84.9 – 93.9) |

* Counted as positive if at least one of the two RDT test strips (NS1, IgM) was positive. Counted as negative if both the NS1 and the IgM strips were negative.

° Counted as positive if at least one of the two tests (dengue RT-PCR and dengue NS1 ELISA) was positive. Counted as negative if both tests were negative.

** Counted as positive if at least one of the three tests (dengue RT-PCR, dengue NS1 ELISA, anti-dengue IgM ELISA) was positive. Counted as negative if all three tests were negative.

^$^ DENV RT-qPCR with Cq<37 were counted as positive and Cq≥37 or no Cqvalue as negative.

^€^ Equivocal results were counted as negative.

**Table S6**: Two by two tables for the calculation of the accuracy of prototype DengueDx and SD Bioline RDTs performed on sera from hospitalized patients in Vientiane Capital, Lao PDR, calculated using qRT-PCR, NS1 and IgM ELISA, alone or in combination, as reference.

|  |  | **Combined RT-PCR, NS1 & IgM ELISA*** | |  |
| --- | --- | --- | --- | --- |
|  |  | **Positive** | **Negative** | **Total** |
| **DengueDx** **RDT (frozen samples) ^$^** | **Positive** | 180 | 19 | 199 |
|  | **Negative** | 96 | 223 | 319 |
|  | **Total** | 276 | 242 | 518 |
| Sensitivity (95%CI): 65.2% (59.3 – 70.8)  Specificity (95%CI):92.1% (88.0 – 95.2)  Positive predictive value: (95%CI): 90.5% (85.5 – 94.2)  Negative predictive value: (95%CI): 69.9% (64.5 – 74.9) | | | | |
| **SD Bioline initial (fresh samples) ^$^** | **Positive** | 213 | 16 | 229 |
|  | **Negative** | 63 | 226 | 289 |
|  | **Total** | 276 | 242 | 518 |
| Sensitivity (95%CI): 77.2% (71.8 – 82.0)  Specificity (95%CI): 93.4% (89.5 – 96.2)  Positive predictive value (95%CI): 93.0% (88.9 – 96.0)  Negative predictive value (95%CI): 78.2% (73.0 – 82.8) | | | | |
| **SD Bioline RDT retesting (frozen samples) ^$^** | **Positive** | 115 | 11 | 126 |
|  | **Negative** | 35 | 116 | 151 |
|  | **Total** | 150 | 127 | 277 |
| Sensitivity (95%CI): 76.7% (69.1 – 83.2)  Specificity (95%CI): 91.3% (85.0 – 95.6)  Positive predictive value (95%CI): 91.3% (84.9 – 95.6)  Negative predictive value(95%CI): 76.8% 69.3 – 83.3) | | | | |
|  |  | **Combined RT-PCR, & NS1 ELISA**^€^ | |  |
|  |  | **Positive** | **Negative** | **Total** |
| **NS1 DengueDx** **RDT (frozen samples)** | **Positive** | 110 | 0 | 110 |
|  | **Negative** | 146 | 263 | 409 |
|  | **Total** | 256 | 263 | 519 |
| Sensitivity (95%CI): 43.0% (36.8 – 49.3)  Specificity (95%CI): 100% (989.6 – 100)  Positive predictive value (95%CI): 100 (96.7 –100)  Negative predictive value(95%CI): 64.3% (59.4 –69.0) | | | | |
| **NS1 SD Bioline initial (fresh samples)** | **Positive** | 187 | 6 | 193 |
|  | **Negative** | 69 | 257 | 326 |
|  | **Total** | 256 | 263 | 519 |
| Sensitivity (95%CI): 73.0% (67.2 – 78.4)  Specificity (95%CI): 97.7% (95.1 – 99.2)  Positive predictive value (95%CI): 96.9% (93.4 – 98.9)  Negative predictive value(95%CI): 78.8% (74.0 –83.1) | | | | |
| **NS1 SD Bioline RDT retesting (frozen samples)** | **Positive** | 91 | 0 | 91 |
|  | **Negative** | 47 | 139 | 186 |
|  | **Total** | 138 | 139 | 277 |
| Sensitivity (95%CI): 65.9% (57.4 – 73.8)  Specificity (95%CI): 100% (97.4 – 100)  Positive predictive value (95%CI): 100% (96.0 – 100)  Negative predictive value(95%CI): 74.7% (67.9 – 80.8) | | | | |
|  |  | **RT-PCR °** | |  |
|  |  | **Positive** | **Negative** | **Total** |
| **NS1 DengueDx** **RDT (frozen samples)** | **Positive** | 100 | 11 | 111 |
|  | **Negative** | 124 | 291 | 415 |
|  | **Total** | 224 | 302 | 526 |
| Sensitivity (95%CI): 44.6% (38.0 – 51.4)  Specificity (95%CI): 96.4% (93.6-98.2)  Positive predictive value (95%CI): 90.1% (83.0 – 94.9)  Negative predictive value (95%CI): 70.1% (65.5 – 74.5) | | | | |
| **NS1 SD Bioline RDT initial (fresh samples)** | **Positive** | 162 | 32 | 194 |
|  | **Negative** | 62 | 270 | 332 |
|  | **Total** | 224 | 302 | 526 |
| Sensitivity (95%CI): 72.3% (66.0 – 78.1)  Specificity (95%CI): 89.4% (85.4 – 92.6)  Positive predictive value (95%CI): 83.5% (77.5 – 88.4)  Negative predictive value (95%CI): 81.3% (76.7 – 85.4) | | | | |
| **NS1 SD Bioline RDT retesting (frozen samples)** | **Positive** | 80 | 11 | 91 |
|  | **Negative** | 40 | 148 | 188 |
|  | **Total** | 120 | 159 | 279 |
| Sensitivity (95%CI): 66.7% (57.5 – 75.0)  Specificity (95%CI): 93.1% (88.0 – 96.5)  Positive predictive value (95%CI): 87.9% (79.4 – 93.8)  Negative predictive value (95%CI): 78.7% (72.2 – 84.3) | | | | |
|  |  | **NS1 ELISA °** | |  |
|  |  | **Positive** | **Negative** | **Total** |
| **NS1 DengueDx** **RDT (frozen samples)** | **Positive** | 108 | 2 | 110 |
|  | **Negative** | 87 | 322 | 409 |
|  | **Total** | 195 | 324 | 519 |
| Sensitivity (95%CI): 55.4% (48.1 – 62.5)  Specificity (95%CI): 99.4% (97.8 – 100)  Positive predictive value (95%CI): 98.2% (93.6 – 99.8)  Negative predictive value (95%CI): 78.7% (74.4 – 82.6) | | | | |
| **NS1 SD Bioline RDT initial (fresh samples)** | **Positive** | 180 | 13 | 193 |
|  | **Negative** | 15 | 311 | 326 |
|  | **Total** | 195 | 324 | 519 |
| Sensitivity (95%CI): 92.3% (87.6 – 95.6)  Specificity (95%CI): 96.0% (93.2 – 97.8)  Positive predictive value (95%CI): 93.3% (88.8 – 96.4)  Negative predictive value (95%CI): 95.4% (92.5 – 97.4) | | | | |
| **NS1 SD Bioline RDT retesting (frozen samples)** | **Positive** | 90 | 1 | 91 |
|  | **Negative** | 17 | 169 | 186 |
|  | **Total** | 107 | 170 | 277 |
| Sensitivity (95%CI): 84.1% (75.8 – 90.5)  Specificity (95%CI): 99.4% (96.8 – 100)  Positive predictive value (95%CI): 98.9% (94.0 – 100)  Negative predictive value (95%CI): 90.9% (85.8 – 94.6) | | | | |
|  |  | **IgM ELISA** ^£^ | |  |
|  |  | **Positive** | **Negative** | **Total** |
| **IgM DengueDx** **RDT (frozen samples)** | **Positive** | 87 | 50 | 137 |
|  | **Negative** | 44 | 341 | 385 |
|  | **Total** | 131 | 391 | 522 |
| Sensitivity (95%CI): 66.4% (57.6 – 74.4)  Specificity (95%CI): 87.2% (83.5 – 90.4)  Positive predictive value (95%CI): 63.5% (54.9 – 71.6)  Negative predictive value (95%CI): 88.6% (85.0 – 91.6) | | | | |
| **IgM SD Bioline RDT initial (fresh samples)** | **Positive** | 67 | 34 | 101 |
|  | **Negative** | 64 | 357 | 421 |
|  | **Total** | 131 | 391 | 522 |
| Sensitivity (95%CI): 51.1% (42.3 – 60.0)  Specificity (95%CI): 91.3% (88.1 – 93.9)  Positive predictive value (95%CI): 66.3% (56.2 – 75.4)  Negative predictive value (95%CI): 84.8% (81.0 – 88.1) | | | | |
| **IgM SD Bioline RDT retesting (frozen samples)** | **Positive** | 52 | 34 | 86 |
|  | **Negative** | 19 | 172 | 191 |
|  | **Total** | 71 | 206 | 277 |
| Sensitivity (95%CI): 73.2% (61.4 – 83.1)  Specificity (95%CI): 83.5% (77.7 – 88.3)  Positive predictive value (95%CI): 60.5% (49.3 –70.8)  Negative predictive value (95%CI): 90.1% (84.9 – 93.9) | | | | |

* Counted as positive if at least one of the three tests (dengue RT-PCR, dengue NS1 ELISA, anti-dengue IgM ELISA) was positive). Counted as negative if all three tests were negative.

**^$^** Counted as positive if at least one of the two RDT test strips (NS1, IgM) was positive. Counted as negative if both the NS1 and the IgM strips were negative.

^€^ Counted as positive if at least one of the two tests (dengue RT-PCR and dengue NS1 ELISA) was positive. Counted as negative if both tests were negative.

° DENV RT-qPCR with Cq<37 were counted as positive and Cq≥37 or no Cq value as negative.

^£^ Equivocal results were counted as negative.
